# Long Term Impact of Conjugate Vaccines on *Haemophilus influenzae* Meningitis: Narrative Review

**DOI:** 10.3390/microorganisms9050886

**Published:** 2021-04-21

**Authors:** Mary Paulina Elizabeth Slack

**Affiliations:** Gold Coast Campus, School of Medicine & Dentistry, Griffith University, Southport, QLD 4222, Australia; m.slack@griffith.edu.au

**Keywords:** *Haemophilus influenzae*, Hib, impact of Hib conjugate vaccine, Hia, NTHi

## Abstract

*H. influenzae* serotype b (Hib) used to be the commonest cause of bacterial meningitis in young children. The widespread use of Hib conjugate vaccine has profoundly altered the epidemiology of *H. influenzae* meningitis. This short review reports on the spectrum of *H. influenzae* meningitis thirty years after Hib conjugate vaccine was first introduced into a National Immunization Program (NIP). Hib meningitis is now uncommon, but meningitis caused by other capsulated serotypes of *H. influenzae* and non-typeable strains (NTHi) should be considered. *H. influenzae* serotype a (Hia) has emerged as a significant cause of meningitis in Indigenous children in North America, which may necessitate a Hia conjugate vaccine. Cases of Hie, Hif, and NTHi meningitis are predominantly seen in young children and less common in older age groups. This short review reports on the spectrum of *H. influenzae* meningitis thirty years after Hib conjugate vaccine was first introduced into a NIP.

## 1. Introduction

*Haemophilus influenzae* is a small, pleiomorphic Gram-negative coccobacillus, which is restricted to humans. It is fastidious in its growth requirement, only growing in culture media supplemented with both X factor (hemin) and V factor (nicotinamide adenine dinucleotide, NAD), for example chocolate agar. *H. influenzae* strains can be differentiated into two major groups: capsulated and non-capsulated strains (generally referred to as non-typeable strains, NTHi). The capsulated strains are further divided into six groups (a to f) based on the chemical structure of their polysaccharide capsules [1]. The most virulent type of *H. influenzae* is type b (Hib) and the major virulence determinant of Hib is its polysaccharide capsule, composed of polyribosyl ribitol phosphate (PRP).

*H. influenzae* colonizes the nasopharynx [2] and to a lesser extent the conjunctivae [3] and genital tract [4,5,6]. The respiratory tract is mainly colonized by *H. influenzae* and to a lesser extent *H. parainfluenzae* [2]. Approximately 80% of individuals carry NTHi strains in the nasopharynx, while 3–5% carry capsulated strains in the upper respiratory tract [7,8]. Spread from one person to another occurs via respiratory droplets or by direct contact with secretions [4].

Before the introduction of Hib conjugate vaccines, Hib was the commonest cause of bacterial meningitis in young children in the United States [9,10], Sweden [11], Iceland [12], the Netherlands [13], and England and Wales [14]. Seventy five percent of Hib meningitis cases occurred in children between the ages of three months and three years [15,16]. The case fatality ratio of Hib meningitis was ~5 to 10% in high-income countries [17].

In 1933, Fothergill and Wright [18] reported that blood from children aged less than two years lacked bactericidal activity against Hib, whereas blood from older children and adults demonstrated bactericidal activity. They speculated that naturally acquired antibodies to Hib were protective and as the mean level of Hib antibodies increased through exposure to the organism, so Hib meningitis incidence declined. The paucity of cases of Hib meningitis in infants aged <two months correlates with the presence of maternal Hib antibodies. This was confirmed by Peltola et al. [19] who demonstrated the incidence of Hib meningitis declined as the mean level of anti-Hib antibodies increased. Studies on un-immunized individuals established a putative short-term correlate of protection against Hib infection of ≥0.15 μg/mL anti-PRP antibodies [20]. Later studies established that an anti-PRP antibody titer of ≥1.0 μg/mL was required for long-term protection [21].

It is now more than three decades since Hib conjugate vaccines were first developed and a variety of vaccine formulations, with a Hib component, are now included in the NIP of almost all countries in the world. Wherever Hib conjugate vaccine has been used the epidemiology of *H. influenzae* meningitis has changed, with Hib meningitis now infrequently seen in young children [22]. However, *H. influenzae* serotype a (Hia) has emerged as a significant cause of meningitis in Indigenous children in North America [23], and non-typeable strains of *H. influenzae* (NTHi) are associated with invasive infections, including meningitis, in neonates, older adults, and other vulnerable patient groups [24]. In 2020, the World Health Organization (WHO) published the document” Defeating meningitis by 2030: a global road map” [25]. The aims of the road map include the reduction of cases and deaths from vaccine-preventable meningitis; introduction of new vaccines; increasing vaccine coverage; and improving surveillance and advocacy. This short review will review the current epidemiology of *H. influenzae* meningitis in the second decade of the twenty first century to assess the progress made to date in achieving the goals set out in this document.

## 2. Method

A PubMed search was performed to identify published papers on the epidemiology of *H. influenzae* meningitis, before and after the introduction of Hib conjugate vaccines, using the terms: (((invasive) AND haemophilus) AND influenzae) AND (“meningitis” OR “nontypable” OR “NTHi” OR “serotype a” OR “serotype b” OR “serotype c” OR “serotype d” OR “serotype e” OR “serotype f” OR “non-b” OR “Hib”) AND (epidemiology OR “burden” OR “risk factor” OR “impact” OR “Hib vaccine” OR “Hib conjugate vaccine” OR “surveillance” OR “review” OR “clinical” OR “outcome” OR “neonate” OR “adult” OR “children”) for papers published between 1985 and 2020. Relevant papers on *H. influenzae* meningitis were reviewed.

## 3. Global Burden of Hib Meningitis before the Introduction of Routine Hib Immunization

Acute meningitis was the most serious presentation of Hib infection, following invasion of the blood stream. Often the child initially developed upper respiratory tract symptoms or otitis media before signs of meningeal involvement [4]. Before the introduction of Hib conjugate vaccines, *H. influenzae* serotype b (Hib) was the commonest cause of bacterial meningitis in young children in the US [9], Sweden [11], Iceland [12], the Netherlands [13], and the UK [14]. The mean annual incidence of Hib meningitis in the US was 54/100,000 (range 19–69/100,000) in children aged < five years and ~120 to 130/100,000 in infants aged < 12 months [26]. Annual rates in Europe, Australia (non-Indigenous children) and South America ranged from <20 to 50/100,000 children aged <5 years [17]. A much higher rate was reported from The Gambia (60/100,000 < 5 years and 297/100,000 < one year of age) [27]. Incidence rates of 282/100,000, 254/100,000, 152/100,000, and 450/100,000 in children aged < five years were reported in Alaska Native [28], White Mountain Apache [29], Navajo Indian [30], and Indigenous Australian [31] children, respectively. A rate of 530/100,000 in children aged <five years was reported in the Keewatin District of Northern Canada, mostly afflicting Inuit children [32].

The majority of cases of Hib meningitis cases occurred in children aged between three months and three years [15,16]. The proportion varied in different parts of the world, with approximately 50%, 40%, and 80% of cases of Hib meningitis occurring in infants aged < 12 months in the US, Europe, and Africa, respectively [17]. In the US and Europe, the peak incidence occurred at eight to nine months of age with less than 10% of cases occurring before the age of six months, and approximately 40% of all cases of Hib meningitis occurred in the first year of life [33]. In Indigenous communities in North America and Australia, and in low and middle income countries (LMICs), the proportion of cases of Hib meningitis occurring in the first six months of life was higher than in industrialized communities [33]. In Australia, the median age of onset of Hib meningitis (and the proportion of cases in the first 12 months of life) in Indigenous and non-Indigenous children was six months (60%) and 15 months (17%), respectively [34]. In The Gambia, 44% and 84% of cases occurred in the first six and twelve months of life respectively [33]. In Alaska Native children, 34% and 67% of cases of Hib meningitis occurred in the first six and twelve respectively [27] (Table 1).

The mean case fatality ratio (CFR) of Hib meningitis ranged from approximately five to ten % in high-income countries to 28% in Africa [17]. Fifteen to 30% of survivors had long-term sequelae, including sensorineural hearing loss, intellectual impairment, epilepsy, cerebral palsy, or hydrocephalus [26,47,48,49,50]. Thirty eight percent of children who survived an episode of Hib meningitis in The Gambia had long-term sequelae [51].

## 4. Hib Vaccines

### 4.1. Plain PRP Vaccine

The first Hib vaccine was a plain polysaccharide vaccine consisting of PRP. It was used in a large field trial in Finland involving 100,000 children aged three months to five years [52]. Although efficacious in children >18 months, it did not induce protective levels of anti-PRP antibodies in children aged <18 months, i.e., those most at risk of Hib meningitis [20,53]. It also failed to have any impact on nasopharyngeal carriage of Hib and so had no impact on transmission [52]. Plain polysaccharide vaccines activate B cells via a T-cell independent pathway, which is poorly developed in children <18 months of age [54]. The antibody response is short-lived, mainly IgM with little isotype switching and no induction of immune memory [55].

### 4.2. Hib Protein-Conjugate Vaccines

In the late 1980s conjugate Hib vaccines were developed in which PRP was covalently linked to a protein carrier. The PRP-protein conjugate induces a T-cell dependent response, which develops at a much younger age in infants, who are able to respond to conjugate vaccines from the age of six to eight weeks [56]. The protein antigen encourages class switching from IgM to IgG via T-helper cells [55]. The IgG generated is predominantly IgG1, which in vitro induces complement-mediated opsonization and bacteriolysis. The antibodies produced are of a higher avidity than those produced by a plain polysaccharide vaccine [55]. Furthermore, PRP-conjugate vaccines have a marked impact on nasopharyngeal carriage of Hib [57]. By reducing nasopharyngeal carriage, transmission of Hib to other susceptible children and adults is interrupted, thereby reducing infection in other non-immunized groups. This is called “herd effect” or “herd protection”.

Four different protein carriers were initially used for Hib conjugate vaccines: tetanus toxoid (PRP-TT), diphtheria toxoid (PRP-D), a non-toxic mutant *Corynebacterium diphtheriae* protein CRM 197 (PRP-CRM) and an outer membrane complex of *Neisseria meningitidis* (PRP-OMP) [58]. The different Hib vaccines were equally immunogenic in adults but elicited different responses in infants <18 months of age. PRP-D was the least immunogenic, generating antibody titers of ≥1.0 μg/mL in approximately 30% of infants after two or three doses [59]. This vaccine was subsequently withdrawn. PRP-OMP vaccine generated antibody titers ≥1.0 μg/mL in 70–80% of infants at two months of age [60] and was the preferred vaccine for use in Indigenous populations in North America and Australia, where there was a very high burden of disease in very young infants [60]. The PRP-TT and PRP-CRM vaccines were similar in their immunogenicity eliciting antibody titers ≥ 1.0 μg/mL after three priming doses [61]. Over time monovalent Hib conjugate vaccines have largely been replaced by combination vaccines, including a bivalent Hib + meningococcus serogroup C vaccine (Hib-MenC), and pentavalent and hexavalent vaccines, where Hib is combined with diphtheria toxoid (D), tetanus toxoid (T), pertussis whole cell (wP) or acellular (aP), and/or hepatitis B (HepB), and/ or inactivated polio vaccine (IPV).

## 5. Introduction of Hib Conjugate Vaccine in National Immunization Programs (NIPs)

Hib vaccine was introduced into the NIP of Finland in 1986 [52], followed by the US in 1987 [62]. In the early 1990s Hib vaccine was added to the NIP in many Western European countries. By 2004, Hib vaccine had been included in the NIP of all European countries and ≥90% high-income countries. The introduction of Hib vaccine into the NIP of LMICs has taken longer, because of several factors. These include a lack of local data on the burden of Hib disease as a result of the difficulties in culturing this fastidious organism, widespread use of antibiotics before collection of blood and cerebro-spinal fluid (CSF) samples for culture and the relatively high cost of the vaccine. In 2004 WHO and the Global Alliance for Vaccines and Immunization (GAVI) sought to address this. Vaccine probe studies [63], in which a randomized controlled trial assesses the difference in incidence of meningitis between children immunized with Hib vaccine and unimmunized children, and the Hib Rapid Assessment Tool (HibRAT) [64] provided data on the burden of Hib meningitis for many LMICs. In 2005, GAVI established the Hib Initiative to accelerate the introduction of Hib vaccine in GAVI-eligible countries [65]. In 2006, WHO recommended the use of Hib conjugate vaccines in all countries [66], thereby allowing GAVI-eligible countries to apply for Hib vaccine without the need to have local data on Hib disease burden. With these measures, the number of countries using Hib vaccine increased from 89/193 (46%) in 2004 to 158/193 (82%) in 2009 [67]. Hib vaccine has now been added to the NIP of all countries in the world, except China, where it is available in the private market and in the Russian Federation, where it is recommended for certain groups of children [68].

## 6. Impact of Hib Conjugate Vaccine on Hib Meningitis

Wherever Hib vaccine has been introduced there has been a significant and sustained decline in Hib meningitis [69,70,71]. In 2000, the global incidence of Hib meningitis was estimated to be 31 (uncertainty range (UR) 16–39) cases/100,000 children aged < five years [72]. The estimated incidence varied considerably by region (Table 2). At that time, the only regions that had widespread use of Hib vaccine were the Americas and Europe. A further analysis of the burden of Hib meningitis in 2000–2015 [73] estimated the global incidence of Hib meningitis had declined to five (UR 2–8) cases/100,000 children aged < five years. There were still regional variations, with the highest estimated incidences in the South East Asian and Western Pacific Regions, which may reflect the lack of introduction of Hib vaccine into some countries in these regions at that time.

By 2015, the burden of Hib meningitis was limited to a small number of countries that had not yet or only recently introduced Hib vaccine in their NIP. In the six years since this study almost all countries have now introduced Hib vaccine and the global burden will have been further reduced. This excellent control depends on maintaining high coverage of Hib vaccine combined with on-going surveillance of all cases of Hib meningitis in all ages of patients.

The estimated global CFR of Hib meningitis in 2000 was 43% (UR 23–55%), ranging from 22% (8–34%) in the Western Pacific Region to 67% (44–75%) in the African Region [72]. By 2015, the global CFR had declined to 19% (7–29%), ranging from 5% (2–8) in Europe and the Western Pacific Region to 61% (20–98%) in the African Region [73].

In 2013, a systematic review of the impact of Hib conjugate vaccine on childhood meningitis mortality, estimated the dose-specific impact (one dose: relative risk, RR = 0.64, 95% CI 0.38–1.06; two doses; RR = 0.09, 95% CI 0.03–0.27; three doses: RR = 0.06, 95% CI 0.02–0.22) [74]. The relative risk (RR) or risk ratio is the ratio of the probability of meningitis in children vaccinated with Hib vaccine to the probability of meningitis in unvaccinated children. This review estimated that three doses of Hib vaccine would prevent 38–43% of childhood meningitis mortality [74].

After the introduction of Hib immunization into several NIPs in the 1990s, the incidence of Hib meningitis declined rapidly [26]. Hib conjugate vaccines have proved to be highly effective in all countries, where there is sustained high coverage of the vaccine [75]. In the US active surveillance of invasive *H. influenzae* disease is undertaken in the Active Bacterial Core Surveillance (ABC) sites, coordinated by the Centers for Disease Control and Prevention (CDC). This surveillance system covers a population of over 42 million in five states and five metropolitan areas across the US [76]. In the 1990s, the rate of bacterial meningitis declined by 55% in the USA following Hib vaccine introduction [77]. Between 1998 and 2007, there were 187 cases of *H. influenzae* meningitis cases identified in the CDC ABC surveillance sites, 9.4% of cases were due to Hib. The overall incidence of *H. influenzae* meningitis declined between 1998–1999 and 2006–2007, from 0.12/100,000 population (95% CI, 0.09 to 0.17) to 0.08/100,000 (95% CI, 0.05 to 0.11) [77]. In 2018, only 38 cases of invasive Hib infection in children aged <five years (incidence 0.19/100,000) were notified throughout the US [78].The number of cases of Hib meningitis was not specified.

In a population-based observational study in Finland, where Hib conjugate vaccine was introduced in 1986, there were 1361 reported cases of bacterial meningitis between 1995 and 2014. Four percent of cases were caused by *H. influenzae* (incidence 0.06/100,000 population) and 92% of the isolates were non-b [79].The median age of *H. influenzae* meningitis was 29 years. From 2004 to 2014 two of 26 *H. influenzae* isolates were Hib [79].

Hib meningitis incidence declined by 72–83% at sentinel hospitals in Pakistan and Bangladesh, respectively, within two years of implementing nationwide Hib conjugate vaccination [80]. In a hospital-based multi-center prospective survey of bacterial meningitis in Turkey from 2015 to 2018, 994 cases of suspected bacterial meningitis in children, aged one month to 18 years, were identified [81]. Three (2.4%) of the 125 culture-positive cases were caused by Hib. Hib conjugate vaccine was introduced in the Japanese NIP in 2013, although Hib vaccine had been available on a voluntary basis since 2008. A nationwide population-based surveillance of invasive *H. influenzae* diseases in children in Japan [82] identified 336 cases of *H. influenzae* meningitis between 2008 and 2017. Between 2008–2012 and 2013–2017 there were 336 and 6 cases of *H. influenzae* meningitis, respectively. No cases of invasive Hib meningitis have been identified since 2014.

Although Hib meningitis has been virtually eliminated in almost all countries with established immunization programs and high vaccine coverage, there have been a few examples of countries that have experienced a re-emergence of invasive Hib infections, including Hib meningitis.

## 7. Resurgence of Hib Meningitis in Some Countries

### 7.1. Resurgence of Hib in the UK

In the UK there was a resurgence in cases in the late 1990s. The UK introduced Hib vaccine in 1992 as a three-dose infant schedule of PRP-TT (at two, three, and four months) with no booster dose in the second year of life, together with a catch-up campaign for all children <five years of age. Hib infections declined rapidly in all age groups through direct and indirect (herd) protection. The incidence of invasive Hib disease in England and Wales declined from 22.9/100,000 children < five years in 1990 to 0.65/ 100,000 in 1998 [83]. From 1999 Hib infections began to increase, especially among toddlers, most of whom were fully immunized. After 1999, the incidence of Hib disease increased to 4.6/100,000 in children aged <five years [84], with many of the infections, including meningitis, occurring in toddlers [55]. Studies established that there was a greater than expected decline in Hib antibodies after primary immunization, which had been initially masked by the catch-up campaign [85,86,87]. The catch-up campaign also contributed to indirect protection by reducing nasopharyngeal carriage. By 1998, all children aged <five years had received three priming doses of Hib vaccine in infancy. A single dose of Hib vaccine administered at the age of 12 months was more immunogenic than three doses given in infancy. Another factor was the use of a less immunogenic Hib combination vaccine with diphtheria, tetanus, and acellular pertussis (DTaP-Hib) in 2000–2001 [84,88]. The resurgence was controlled by the re-introduction of a whole-cell pertussis-containing Hib vaccine (DTwP-Hib) in 2002, an Hib booster campaign for toddlers in 2003, and the introduction of a routine 12-month Hib booster in 2006 [89,90].

Since that time Hib infections, including meningitis, have remained at a very low level in the UK, A review of invasive Hib infections in England and Wales, between 2009 and 2012, identified only 14 cases in 2012 [22]. Hib incidence was 0.06/100,000 (two cases) in children aged <five years [22]. Most of the cases that occurred over those four years were in adults (73%), many of whom had underlying comorbidities and presented with pneumonia (56%) [22]. The Hib-associated case fatality rate was 9.4% (10/106 cases) [22]. There were 20 cases (18.9%) of meningitis: ten in children aged < one year; five in children aged one to five years, two in adults aged 20 to 44 years, two in adults aged 45 to 64 years and one case in an older adult aged ≥65 years [22]. There was only one death in the vaccine-eligible age cohort: a child with Hib meningitis who was partially vaccinated and had a complement deficiency [22]. Hib meningitis is now uncommon in the UK.

The current Hib vaccination program in the UK is hexavalent vaccine (DTaP-Hib-HepB-IPV) administered at two, three, and four months, with a 12-month booster dose of Hib-MenC vaccine [91]. The number of cases of invasive Hib infection is at a very low level, with only five cases of invasive Hib disease (cases that were meningitis not specified) in the vaccine eligible population in 2017–2018 [92].

### 7.2. Resurgence of Hib in South Africa

South Africa introduced Hib conjugate vaccine (PRP-TT) in 1999 as an early accelerated schedule of three doses at six, ten, and fourteen weeks without a booster dose in the second year of life [93]. The number of cases of invasive Hib infection initially declined, but from 2005 increasing number of cases in fully vaccinated children were detected [94]. Despite high vaccination coverage the detection rate of invasive Hib infection in children aged < five years increased from 0.7/100,000 in 2003 to 1.3/100,000 in 2009 (*p* < 0.001), and 135/263 (51%) of cases in children with known vaccination status were Hib vaccine failures [93]. From 2003 to 2009 the surveillance program (GERMS) identified 349 cases of invasive Hib infection in children aged <five years, of which 211 (60%) presented as meningitis [94] with a CFR of 19%. Fifty-five% of the children, where HIV status was documented, were HIV negative. Following the addition of a booster dose of Hib vaccine in 2009, as a pentavalent vaccine (DTaP-Hib-IPV) the incidence of invasive Hib declined [92]. In 2018, GERMS identified 327 cases of invasive *H. influenzae* infection, of which 201 were available for typing. Seventeen percent (34/201) were Hib, of which eight cases presented with meningitis [95].

### 7.3. Resurgence of Hib in the Gambia

The Gambia introduced Hib vaccine in 1997. Before the Gambia introduced routine Hib vaccination, Hib meningitis incidence was 297/100,000 in infants <one year of age and 60/100,000 in children aged <five years [27]. The Gambia used a three-dose primary series of PRP-TT Hib vaccine, administered at two, three, and four months without a booster dose. For 14 years invasive Hib disease was well controlled in this country with consistently high coverage, low carriage rates and high levels of protective antibodies [96]. On-going surveillance in eastern Gambia identified an increase in Hib infections between 2011 and 2013, with 17 cases of invasive Hib infection, including 14 cases of Hib meningitis [97]. Although the reason for this re-emergence is not entirely clear, it does emphasize the importance of on-going surveillance.

### 7.4. Is a Booster Dose of Hib Vaccine Needed?

Although these instances where invasive Hib infections have emerged were in countries using a three dose primary series of Hib vaccine without a booster dose, Kenya and most LMICs use this schedule with no evidence of a resurgence of invasive Hib cases [98]. A three dose primary series of Hib vaccine without a booster dose is recommended by WHO [66]. A meta-analysis of 20 RCTs, conducted in 15 countries comparing different Hib vaccination schedules (3 + 0, 3 + 1, and 2 + 1) and different intervals between the primary, and the primary and booster doses, concluded that there was no difference between the schedules in terms of preventing invasive Hib disease, clinical effectiveness or immunologic response. All of the schedules protected against Hib infection and local epidemiology should determine the schedule, with three doses in the first six months of life being more appropriate where the greatest burden of Hib infection is in the first year of life, as in sub-Saharan Africa. Where the burden of infection occurs at a later age, the third dose could be given in the second year of life. In countries like the UK, where Hib infections resurged with a 3 + 0 schedule, a booster in the second year of life may be required [99]. Children who are HIV infected may require a booster dose of vaccine [100].

## 8. Current Burden of *H. influenzae* Meningitis

When Hib vaccine was first introduced there were concerns that Hib meningitis might be replaced by infections caused by other serotypes of *H. influenzae*. This has generally not happened, except in the Indigenous communities of North America, where *H. influenzae* serotype a (Hia) has emerged as a significant pathogen [23,101]. There has also been a slight increase in infections, including meningitis, caused by Hie and Hif in Europe [102,103]. Invasive infections caused by non-typeable strains of *H. influenzae* (NTHi) have increased significantly in many regions of the world [103,104].

## 9. Meningitis Due to Non-b Serotypes of *H. influenzae*

### 9.1. Meningitis Due to Serotype a (Hia)

Before the introduction of Hib vaccine, invasive *H. influenzae* serotype a (Hia) disease was very uncommon, although Hia was responsible for 12% of cases of bacterial meningitis in young children in Papua New Guinea before the introduction of Hib immunization [105,106]. Over the last two decades Hia has emerged as a significant pathogen, particularly in Indigenous populations in North America [23]. High incidences of Hia infection have been reported in Alaska Native, American Indian, and Canadian Inuit children [29,107,108,109,110]. In 2011, a population-based study in 12 Canadian pediatric tertiary care centers reported an Hia incidence of 418.8/100,000 in Inuit children aged < five years in the Keewatin region [111]. Hia is the second most virulent capsular serotype of *H. influenzae* [112] and can cause meningitis, pneumonia, septic arthritis, and bacteremia [23]. Most Hia infections occur in children aged six months to two years [23]. Between 1998 and 2003 38/76 (50%) of cases of Hia infection identified in Navajo and White Mountain Apache children presented with meningitis [107]. Hia meningitis was the commonest presentation in Indigenous children in the North American Arctic and Northern Canada [108,110]. Hia has also emerged as a significant pathogen in Utah and North and South Dakota [113,114,115,116,117]. In a study in Utah from 1998 to 2008, 28% of all invasive disease in children aged < five years was due to Hia, and 18% due to Hib. Fifty percent of the Hia cases presented as meningitis [115]. Hia infections in these states were not exclusively in American Indian children. Hia infections have also been reported from Brazil [118,119,120]. The case fatality rate of Hia meningitis was 14% in Brazil [120], 16% in Northern Canada [110], and 6% in the North American Arctic [112]. Hia has also been reported in Italy [121] and England [122] but there were no cases of meningitis in these reports of infections, which predominantly occurred in adults. Hia meningitis in a 10 month old infant and a 3 year old child was reported from Saudi Arabia [123]. The emergence of Hia as a significant cause of invasive infections in certain populations has prompted the development of an Hia conjugate vaccine [124].

### 9.2. Meningitis Due to Serotypes e and f (Hie and Hif)

There has also been increasing recognition of cases of meningitis caused by Hie and Hif [102]. Between 2001 and 2010 the year on year incidence of Hie and Hif infections in England and Wales increased by 7.4% and 11.0% respectively [100]. In 2009–2010, the incidences of Hie and Hif infections were 0.03/100,000 persons and 0.09/100,000 persons, respectively, with the highest rates being seen in infants and older adults [102]. Nine of 10 cases occurring in infants aged <one year presented with meningitis (three Hie, six Hif). All of the infants with Hif meningitis survived, but one child with Hie meningitis died, one had severe bilateral sensorineural deafness and one developed seizures [102]. Meningitis was a less common presentation in older children and adults, with three cases of Hie meningitis (one child aged one to four years, one child aged five to 14 years, one adult aged 15–64 years) and four cases of Hif meningitis (one in a child aged one to four years, three in adults aged 15–64 years). The case fatality rates of Hie and Hif meningitis were 14.3% and 0%, respectively. In this study Hie meningitis was associated with more complications and a higher case fatality rate.

Whittaker et al. [125] analyzed reports of invasive *H. influenzae* infection reported by 12 European countries to the European Centre for Disease Prevention and Control (ECDC) between 2007 and 2014. Five hundred and ninety-six cases of meningitis were reported, representing 9% of all infections. Sixty percent and 40% of infants aged <one year with Hie or Hif infection were reported to have meningitis [125]. National surveillance in Germany between 2001 and 2016 identified 351 cases of capsulated *H. influenzae* invasive infection: 241 cases of Hif, 45 cases of Hie, seven cases of Hia, and 58 cases of Hib(126). Forty cases of Hif infection were in children aged < four years with 40% of these cases presenting as meningitis. There were 185 cases of Hif infection in adults aged ≥ 40 years with meningitis accounting for 15% [126]. Hif meningitis has also been reported in the United States [127,128,129] and in Sweden [130].

### 9.3. Meningitis Due to Non-Typeable H.influenzae (NTHi)

Since the introduction of Hib vaccine, NTHi infections have emerged as the most common cause of invasive *H. infuenzae* infection in many parts of the world, where surveillance has been undertaken [104,113,125,126,127,128,130,131,132,133,134,135,136,137,138,139]. The highest burden of NTHi infections is seen in neonates, children aged <one year, pregnant/post-partum women, and in older adults (≥65 years) [104]. The clinical presentation varies by age, with meningitis more commonly seen in older infants and children and pneumonia more common in older adults [104].

Over a five year period (2009–2013), there were 115 cases of neonatal invasive NTHi infection in England and Wales (incidence 4.1/100,000; 95% CI 3.4–5.0) [24]. The incidence was significantly higher in premature babies (28.4/100,000; 95% CI 22.8–35.0) compared to those born at term (0.9/100,000; 95% CI 0.6–1.4) and increased exponentially with increasing prematurity. For infants born at <28 weeks’ gestation the incidence was 342/100,000 (95% CI, 234–483). Most cases (110/115, 96%) presented within 48 h of birth. Although most of the infants developed a bacteremia, 11 (10%) presented with meningitis. One infant with meningitis died and five (50%) developed long-term sequelae [24].

Active surveillance for invasive *H. influenzae* disease in the US ABC surveillance sites from 2009 to 2015, reported that invasive NTHi infections had the highest incidence (1.22/100,000) [113]. Among 317 cases of invasive *H. influenzae* infection in children aged <one year, 25.1% presented with meningitis. One hundred and ninety six of 294 (66.7%) invasive infections (where the serotype was known) in this age group were due to NTHi. Although the serotyping of the meningitis cases was not reported it is probable that they included cases of NTHi meningitis.

Between 2001 and 2008, there were 396 cases of invasive NTHi infection documented by the Netherlands Reference Laboratory for Bacterial Meningitis [134]. Overall, the most common presenting clinical syndrome was invasive pneumonia (190/396, 48%) followed by bacteremia (75/396, 19%). Fifty-seven (14%) of the cases presented with meningitis. Among children aged seven weeks to <five years 28/60 (47%) of cases were meningitis. Nationwide active surveillance in Germany between 1998 and 2005 identified 70 cases of invasive NTHi infection. The median age of presentation was 26 months (0–73 months) and 34% presented with meningitis [135]. Thirty eight percent of children with NTHi meningitis had predisposing conditions, including prematurity, immunodeficiency, and Down’s syndrome [135]. In a study from England [131] 26% of children who survived NTHi meningitis suffered long-term sequelae, including deafness, seizures, and hydrocephalus [131]. The case fatality rate of NTHi meningitis is similar to that of Hib meningitis [131].

## 10. Conclusions

Hib conjugate vaccine has been a remarkable success story, reducing the incidence of Hib meningitis to a very low level in countries with a well-established Hib immunization program and sustained high vaccine coverage [140]. There has been considerable progress in achieving the elimination of *H. influenzae* meningitis, but more still needs to be done. Cases of Hib meningitis do still occur, in unimmunized or partially vaccinated children, and as rare instances of true Hib vaccine failures. In 2015, Wahl et al. [73] estimated that there were still 12,900 cases (UR 6400 to 21,500) of Hib meningitis globally. Since then, Hib vaccine has been introduced into the NIP of almost all countries, including India and Thailand, except for China and the Russian Federation (where Hib vaccine is recommended for certain risk groups). Every child in the world should be offered Hib vaccine and vaccine coverage needs to be maintained at a high level in all countries. Hia has emerged as a significant cause of meningitis in Indigenous populations of North America, potentially requiring the use of Hia conjugate vaccine in these high-risk populations. Hie, Hif, and NTHi have also been associated with cases of meningitis. The changing epidemiology of *H. influenzae* meningitis emphasizes the importance of on-going surveillance. Epidemiologic and microbiologic surveillance should be comprehensive, covering all ages and all types of *H. influenzae*. Accurate typing of strains, using molecular methods combined with clinical ascertainment of clinical presentation, underlying risk factors and outcome should be undertaken to fully document these changes. Considerable progress in achieving the elimination of *H. influenzae* meningitis has been made, but more still needs to be done. 

## Figures and Tables

**Table 1:** Incidence of Hib meningitis before the introduction of routine Hib conjugate vaccination.

Region	Hib Meningitis (Cases/100,000 Children < 5 Years of Age)
USA	54
North America (Indigenous)	152–530
Europe	23–31
Israel	18
The Gambia	60
Australia and New Zealand (non-Indigenous)	25–34
Australia and New Zealand (Indigenous)	450
Latin America	35
Asia	25
Mongolia	28

Data derived from: USA [9,10]; North American (Indigenous) [28,29,30,35]; Europe [36,37,38,39,40]; Israel [41]; The Gambia (Reference [27]; Australia and New Zealand (non-Indigenous) [34,42]; Australia and New Zealand [31,43]; Latin America [44]; Asia [45]; and Mongolia [46].

**Table 2:** Estimated incidence and case fatality ratio of Hib meningitis (with uncertainty estimates) by WHO region in 2000 and 2015.

	Global	African Region	Region of the Americas	Eastern Mediterranean Region	European Region	South East Asia Region	Western Pacific Region
**2000 Estimates**							
**Incidence**	31 (16–39)	46 (31–52)	25 (16–30)	24 (14–35)	16 (12–22)	27 (11–38)	34 (12–48)
**CFR**	43% (23–55%)	67% (44–75%)	28% (15–36%)	44% (26–62%)	27% (17–41%)	44% (17–62%)	22% (8–34%)
**2015 Estimates [73]**							
**Incidence**	5 (2–8)	2 (1–3)	0 (0–0)	1 (0–1)	3 (1–5)	8 (3–12)	11 (6–18)
**CFR**	19% (7–29%)	61% (20–98%)	30% (7–51%)	54% (16–89%)	5% (2–9%)	32% (12–49%)	5% (2–8%)

Data are estimates (uncertainty range) Incidence is /100,000 children aged <5 years. CFR: case fatality ratio. Data derived from: Watt et al. [61] and Wahl et al. [62].

## Data Availability

Data available in a publicly available repository (PubMed).
